# Segregation and pattern formation in dilute granular media under microgravity conditions

**DOI:** 10.1038/s41526-016-0009-1

**Published:** 2017-01-05

**Authors:** Eric Opsomer, Martial Noirhomme, Nicolas Vandewalle, Eric Falcon, Simon Merminod

**Affiliations:** 1 Université de Liège, GRASP, Unité de Recherche CESAM, Liège, B-4000 Belgium; 2 Université Paris Diderot, Sorbonne Paris Cité, MSC, UMR 7057 CNRS, Paris, F-75013 France

## Abstract

Space exploration and exploitation face a major challenge: the handling of granular materials in low-gravity environments. Indeed, grains behave quite differently in space than on Earth, and the dissipative nature of the collisions between solid particles leads to clustering. Within poly-disperse materials, the question of segregation is highly relevant but has not been addressed so far in microgravity. From parabolic flight experiments on dilute binary granular media, we show that clustering can trigger a segregation mechanism, and we observe, for the first time, the formation of layered structures in the bulk.

During the past decades space exploration and exploitation has remained in the spotlight of the scientific community and industry. Indeed, landing probes on distant planetoids, extracting rare earth elements on asteroids and 3D printing infrastructures on the Moon is as exciting as it could be lucrative.^1^ However, difficulties arise when it comes to the handling of regolith, the granular materials present on the surface of dusty celestial bodies.^2^ The physical ingredients of granular physics in space are straightforward: dissipative collisions, cohesion and electrostatic interactions for fine grains. For inelastic particles, i.e. with only dissipative collisions, a clustering of the granular material can occur. The latter phenomenon has been studied in microgravity since the nineties and was observed experimentally for the first time during a sounding rocket mission.^3^ Since then, many numerical and experimental studies in low gravity^4–7^ were realised mainly with mono-disperse systems. However, recent numerical studies^8^ indicate that the poly-dispersity of the granular media impacts strongly on its behaviour and may lead to segregation in zero *g* even though the usual mechanisms responsible for this phenomenon rely on the presence of gravity.

Here, we present novel experimental results from the European Space Agency’s parabolic flight campaign PFC64 exhibiting spectacular pattern formation within a binary granular system under microgravity conditions. Our experiments were performed within the framework of the VIP-Gran instrument, whose setup consists in a 45 × 30 × 5 mm^3^ rectangular box in which two opposing walls (30 × 5 mm^2^) act as pistons. They oscillate sinusoidally in anti phase along the longitudinal axis (45 mm) of the box with a typical amplitude of 3 mm and a typical frequency of 20 Hz. We studied four different granular loadings composed of *N*_s_ small and *N*_l_ large bronze beads with respective diameters *d*_s_ = 1 mm and *d*_l_ = 2 mm. Both species have respective masses *m*_s_ = 4.8 mg and *m*_l_ = 38.4 mg. The restitution coefficient for the different types of collisions were not measured experimentally. However, given the low grain velocities encountered in our experiment, a value of *ε* = 0.9 can be assumed for bronze-bronze interaction^9^.

The initial loading of the cell is 80 large beads; we then inject sequentially two slots of 500 small particles and a slot of 150 large ones. This leads to volume fractions *ϕ* between 3% and 17% corresponding to low-density regimes. The parameter *ϕ* is defined as follows:

1 $$\phi ={\phi }_{{\rm{s}}}+{\phi }_{{\rm{l}}}=\pi \frac{{N}_{{\rm{s}}}{d}_{{\rm{s}}}^{3}}{6V}+\pi \frac{{N}_{{\rm{l}}}{d}_{{\rm{l}}}^{3}}{6V},$$

where *V* is the volume of the cell. While shaking the system in microgravity, four different dynamics are observed: First, the mono-disperse system exhibits typical dilute granular gas dynamics. Particles move erratically and are equally distributed everywhere in the container (Fig. 1a). After the injection of 500 small particles, clustering occurs. However, given the dissipative nature of the collisions, the large beads cool down more rapidly than the small ones and migrate toward the centre of the bulk (Fig. 1b). An analogous segregation phenomenon, relying on the gradient of granular fluctuation energy, has been observed in the case of a collisional granular shear flow.^10^ If one continues to increase *N*_s_, small particles also contribute to the cluster, but the kernel of the bulk remains mainly composed of large grains (Fig. 1c). Once the additional 150 large beads are added into the system, the structure of the bulk changes dramatically. More and more small beads contribute to the cluster and force the large ones towards the interface with the surrounding gas (Fig. 1d). Finally, the system exhibits a complex pattern constituted of successive layers with alternatively high concentrations of small and large particles. Although this particular behaviour was theoretically predicted,^11^ it is the first time that such pattern is experimentally observed in microgravity.

**Fig. 1:**
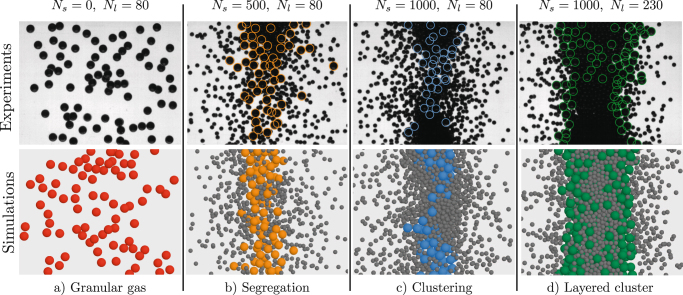
Snapshots (*bottom view*) during experiments (*top*) and simulations (*bottom*). Large beads are highlighted with coloured circles. In the system with 80 (**a**) large beads, granular gas is observed. After the addition of 500 (**b**) and 1000 (**c**) small particles, segregation occurs and the large beads gather in the central bulk. Then 150 additional large grains (**d**) are injected and a complex pattern arises. The sinusoidal forcing is along the horizontal axis

In addition to our experiments, we realised Soft Sphere Discrete Element Method simulations of our setup and established a (*ϕ*_s_, *ϕ*_l_) phase diagram presented in Fig. 2. Normal contact forces are modelled via a linear spring-dashpot. Tangential friction forces are proportional to the sliding velocity and are bounded via Coulomb’s criterion. For all types of contacts, the restitution coefficient is fixed to *ε* = 0.9 and the friction coefficient to *μ* = 0.4. As described in Opsomer et al.^8^ the clustering of the individual granular species can be detected via a statistical uniformity test and the frontiers between the different dynamical regimes can be drawn accordingly. Granular gas is found in the lower left corner of the diagram (red). Segregation is observed for a wide range of fillings in the central region of the diagram (orange). Clustering of both particle species is encountered for high numbers of small grains, in the lower right corner (blue). Stripy patterns can only be found for the highest filling values, in the upper right corner (green). Snapshots of all presented experiments as well as corresponding simulations are compared in Fig. 1. Large beads are highlighted with coloured circles. Filling increases from left to right and the sinusoidal driving is along the horizontal direction.

**Fig. 2:**
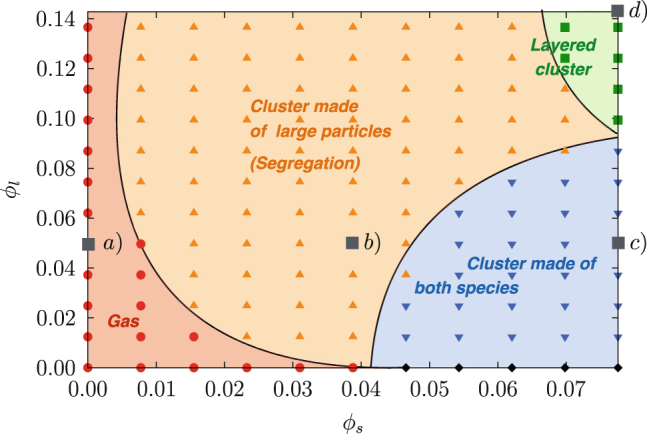
Phase diagram obtained via numerical simulations by varying filling concentrations of *ϕ*_s_ and *ϕ*_l_. Clustering of the individual species is detected using a statistical test of uniformity of the particle’s positions in the cell. Different colours and symbols are used depending on the detected regime. *Solid curves* serve solely as guides. Large *square* symbols correspond to experimental conditions Fig. 1

We observed, for the first time, segregation coupled to complex pattern formation in a driven binary mixture of grains under microgravity conditions. Our results are intriguing since the usual mechanisms responsible for segregation, such as convection and percolation, all rely on the presence of gravity. Our work could lead to a better understanding of the surface properties of dusty planetoids and enhance the handling and transport procedures of granular materials in space.
